# Supplementary material for: Bioluminescent-Inhibition-Based Biosensor for Full-Profile Soil Contamination Assessment
Source: Biosensors (Basel). 2022 May 19;12(5):353. doi: 10.3390/bios12050353 (PMC9138560; doi:10.3390/bios12050353)
Supplement: Supplementary file 1 [file biosensors-12-00353-s001.zip › biosensors-1721558-supplementary.pdf]

Supplementary Materials 1

Table S1—Profile structure, chemical properties and value of residual luminescence of agricultural soils.

Horizon index and thickness	Sampling depth, cm	Humus. %	pH_{KCl}	LHSs, mg C/100g				I/I0	(I/I0) av.
				sum	HA	FA	HA:FA	%	
Humus-accumulative order. Clay-illuvial agrochernozem, moderately deep, highly humous, heavy loamy, on clay. Agricultural grassland (perennial grasses).									
PU. 0-20	0-10	5.78	5.76	431	242	189	1.28	24.2	42.3
	10-20	5.35	5.70	388	210	178	1.18	29.2	
AU. 27-43	27-43	4.92	5.57	336	147	189	0.78	35.4	
ABI. 43-55	43-55	2.47	5.35	135	41	94	0.44	45.5	
BI. 55-120	55-75	1.52	5.06	82	31	51	0.61	63.7	
	75-120	1.09	4.47	40	20	20	1.00	55.7	
Humus-accumulative order. Agrochernozem, minor soil, highly humous, heavy loamy, on carbonate heavy loam. Ten-year fallow lands.									
AU (PU). 0-26	0-12	6.31	5.44	304	168	136	1.24	36.9	67.0
	12-26	5.99	5.64	304	179	125	1.43	38.0	
ABI. 26-52	35-40	2.37	5.77	50	25	25	1.00	75.0	
BCA. 52-140	70-80	0.77	7.19	19	9	10	0.90	85.5	
	115-125	0.77	7.25	19	9	10	0.90	99.7	
Humus-accumulative order. Agrochernozem, minor soil, rich in humus soil, heavy loamy, on carbonate loam. Virgin land.									
AU. 0-34	0-7	9.40	5.83	568	348	220	1.58	32.7	63.2
	15-30	7.48	5.92	283	115	168	0.68	56.9	
ABI. 34-45	34-45	2.26	5.52	103	31	72	0.43	49.7	
BI. 45-67	50-65	1.62	5.62	82	20	62	0.32	64.2	
BCA. 67-120	72-82	1.41	7.11	50	10	40	0.25	95.5	
	100-110	1.20	7.29	40	10	30	0.33	80.0	
